# Qualitative MRI features in the differentiation between primary and secondary CNS lymphoma

**DOI:** 10.1007/s00234-025-03594-1

**Published:** 2025-03-17

**Authors:** Yusuf Kenan Cetinoglu, Kazım Ayberk Sinci, Merve Horoz, Fazıl Gelal

**Affiliations:** 1 Department of Radiology, Health Science University Dr. Behçet Uz Children Disease and Surgery Training and Research Hospital, İzmir, Türkiye; 2 Department of Radiology, Kanuni Sultan Suleyman Education and Research Hospital, İstanbul, Türkiye; 3 Department of Radiology, Faculty of Medicine, İzmir Democracy University, İzmir, Türkiye; 4 Department of Radiology, İzmir Kâtip Çelebi University Atatürk Training and Research Hospital, İzmir, Türkiye

**Keywords:** Primary CNS lymphoma, Secondary CNS lymphoma, VASARI, Notch sign, MRI

## Abstract

**Purpose:**

Differentiating between primary CNS lymphomas (PCNSL) and secondary CNS lymphomas (SCNSL) remains a challenge in imaging. The aim of this study was to differentiate histopathologically-proven PCNSL and SCNSL by using 25 qualitative VASARI and five other MRI features.

**Methods:**

MRIs of 31 cases (19 PCNSL and 12 SCNSL) obtained between January 2010 and February 2022 were retrospectively reviewed. Two blinded readers independently evaluated images without knowledge of clinical data or whether CNS lymphoma was primary or secondary. The findings of each reader were recorded to assess interreader agreement. The results of two readers were evaluated by a senior neuroradiologist to reach a consensus. A statistical analysis was performed on the collected data.

**Results:**

Most VASARI features showed no statistically significant differences between the two groups, except for two features. Tumor location exhibited a statistically different distribution between PCNSL and SCNSL groups (*p* = 0.036). Proportion of edema was greater in the PCNSL group compared to the SCNSL group (*p* = 0.049). Among other MRI features, infratentorial involvement was more frequent in the SCNSL group (*p* = 0.014), while notch sign was more commonly detected in the PCNSL group (*p* = 0.027). Inter-reader agreement for VASARI features ranged from moderate to almost perfect, and for other MRI features, it ranged from fair to almost perfect.

**Conclusion:**

Despite the challenges in distinguishing imaging features of PCNSL and SCNSL; frontal lobe location, a higher proportion of edema and the presence of a notch sign may indicate PCNSL, while infratentorial involvement may suggest SCNSL.

## Introduction

Central nervous system (CNS) lymphomas are divided into primary central nervous system lymphoma (PCNSL) and secondary central nervous system lymphoma (SCNSL). In PCNSL, which accounts for 2–4% of all brain tumors, the disease is limited to the brain, leptomeninges, spinal cord, or eye [1, 2]. Unlike PCNSL, SCNSL is characterized by CNS involvement of systemic lymphoma.

Contrast-enhanced magnetic resonance imaging (MRI) is the modality of choice for the diagnosis, prognosis, and treatment response of CNS lymphomas. Few studies in the literature have investigated the differentiation of PCNSL and SCNSL based on imaging findings [3–7]. None of these studies found a significant difference in MRI findings between PCNSL and SCNSL. The initial diagnostic workup for managing PCNSL and SCNSL differs as it entails searching for the primary site of origin. At the initial diagnosis, approximately 8% of cases initially evaluated as PCNSL were reported to have occult systemic lymphoma, hence imaging with whole-body CT, ^18^FDG-PET, and testicular ultrasonography are recommended for extra-CNS disease assesment [8–10]. PCNSL has a poorer prognosis compared to localized large-cell lymphomas outside the CNS. For most patients with SCNSL, CNS involvement is not the primary cause of death. The survival rate depends on systemic disease rather than CNS involvement [11, 12]. Therefore, it may be beneficial to patient management to make the differentiation between PCNSL and SCNSL by MRI at an earlier time.

The VASARI (Visually AcceSAble Rembrandt Images) feature guide was developed by experienced neuroradiologists in 2008 to improve accuracy, reproducibility, and standardization in the evaluating MR images of primary brain tumors. This guide has been further developed in subsequent years by The Cancer Genome Atlas Glioma Phenotype Research Group (2020) [13]. It includes 25 imaging features that evaluate lesion localization, lesion morphology, contrast enhancement, changes in the lesion periphery, and the presence of imaging features such as bleeding, cyst, satellite lesion, and calvarial remodelling.

While the VASARI feature set has mostly been applied to gliomas and, in one study, to distinguish between brain metastases and glioblastoma [14–18], its use in CNS lymphoma has not been evaluated to our knowledge. This study aims to evaluate differentiating primary and secondary CNS lymphomas on MRI by using twenty-five VASARI features and five other qualitative MRI features including infratentorial involvement, coexistence of both infratentorial and supratentorial involvement, enhancement pattern, notch sign and butterfly sign.

## Materials and methods

Institutional Review Board approval was obtained from the Non-Invasive Clinical Research Ethics Committee of İzmir Kâtip Çelebi University with the application number 2023-GOKAE-0411 in this retrospective study.

### Patient selection

**Fig. 1:**
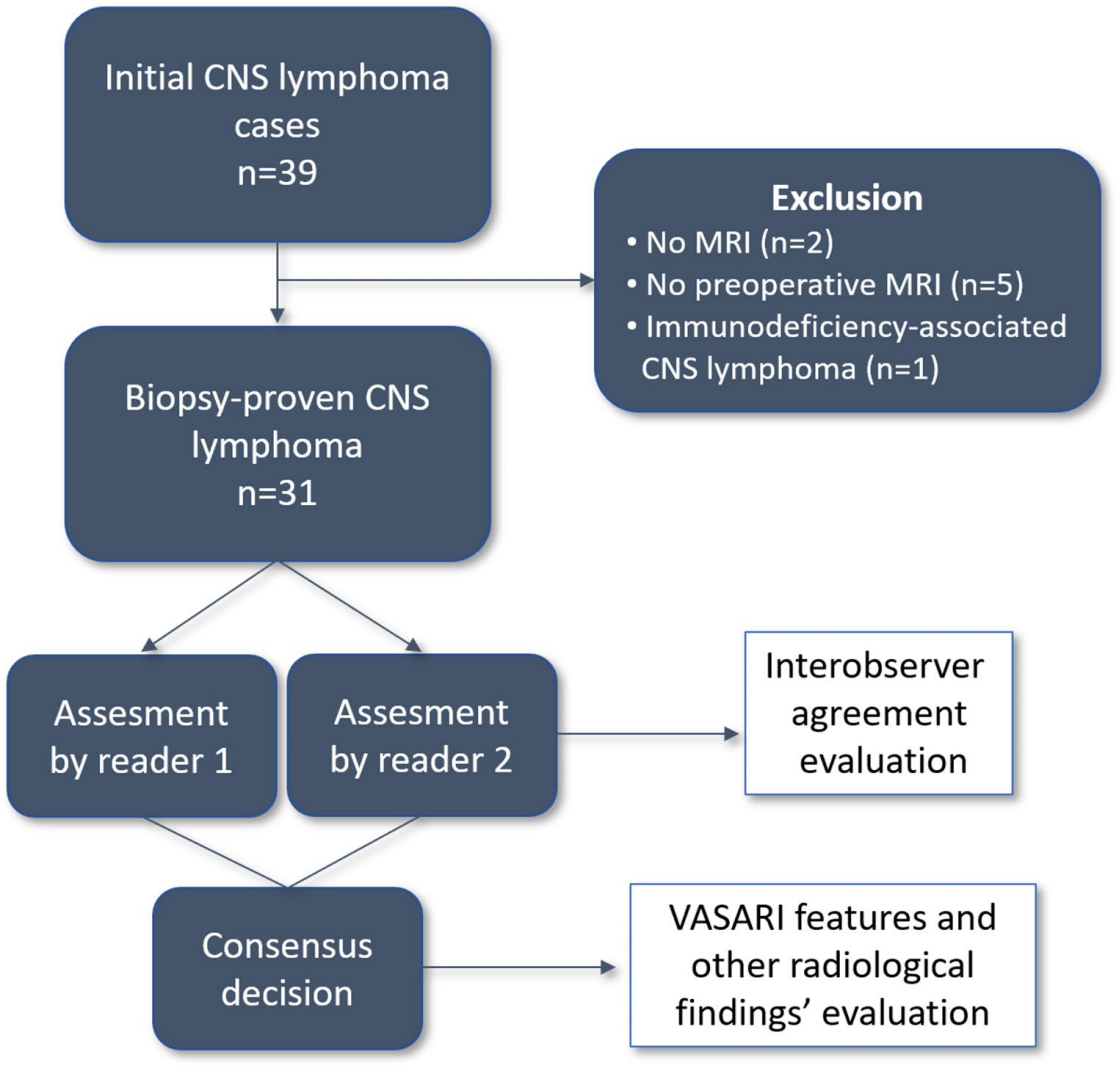
Flow chart of the study

We retrospectively reviewed our center’s database for cases with biopsy-proven CNS lymphoma, covering the time period from January 2010 to February 2022. Initially, the MR images of 39 histopathologically confirmed CNS lymphoma cases with parenchymal involvement were included in the study. Five patients without preoperative MRI and two patients without any MRIs in our institute’s Picture Archiving and Communication Systems (PACS) were excluded. In addition, an HIV-positive case was excluded due to the distinct differences in imaging characteristics of immunodeficiency-associated CNS lymphoma compared to typical CNS lymphomas [19]. The initial contrast-enhanced brain MR images of the remaining 31 cases with a confirmed biopsy-proven CNS lymphoma were evaluated (Fig. 1). MR imaging protocols were different due to the wide time span of the study. MR images were acquired on three different 1.5 T MR scanners (Philips Medical Systems, Siemens Medical Systems, and GE Medical Systems). The imaging protocol included T1-weighted spin-echo (TR/TE: 250–650/11–15 ms, matrix: 224–384 × 205–235), T2-weighted turbo spin-echo (TR/TE: 3537–5210/76–110 ms, matrix: 288–384 × 245–352), T2-weighted fluid attenuated inversion recovery (FLAIR) (TR/TE: 6000–8800/86–120 ms, TI: 2000–2133, matrix: 256–320 × 159–256), post-contrast T1-weighted spin-echo imaging in all three orthogonal planes (TR/TE: 12–433/2–15 ms, slice thickness: 1–5 mm, matrix: 256–384 × 179–269). Diffusion-weighted imaging (DWI) was applied in 21 patients with parameters as follows: TR/TE: 5970–6130/13–42, b value: 0–1000 s/mm^2^, matrix: 128–168 × 128–168. Susceptibility Weighted Angiography (SWAN) or Susceptibility Weighted Imaging (SWI) of 23 patients was used to evaluate the presence of hemorrhage, which is one of the features in the VASARI guide. In the remaining eight patients, the presence of hemorrhage was evaluated with conventional MR images.

### Image analysis

**Fig. 2:**
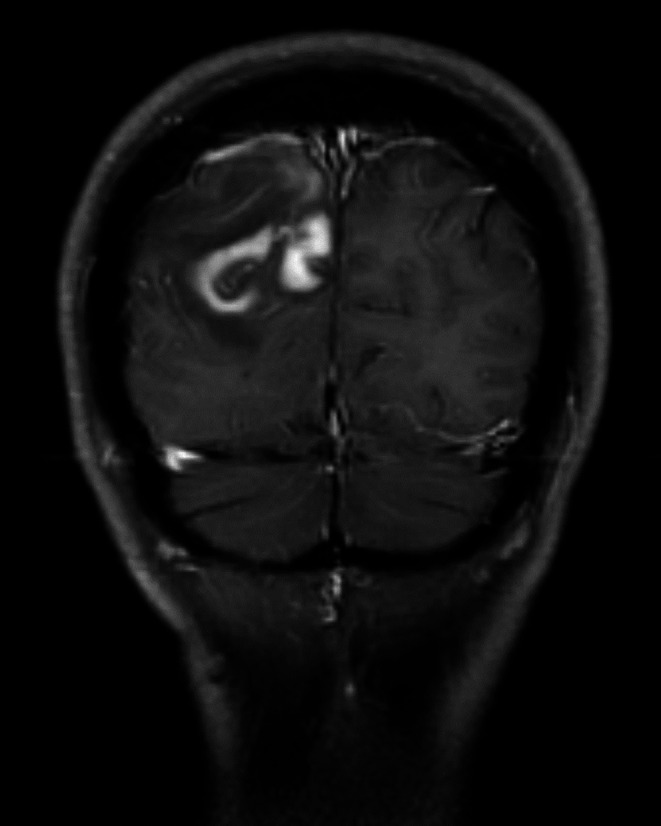
Notch sign on contrast-enhanced coronal T1W image of a 48-years-old patient with PCNSL. The tumor exhibits a depression towards the parietal cortex without causing invasion due to the pliability of the tumor

The patients who had involvement only in CNS were grouped as PCNSL lymphoma; and those with both CNS and extra-CNS organ involvement identified through bone marrow biopsy, whole-body CT, and/or PET/CT were grouped as SCNSL. Then, various MRI findings in each of these groups were statistically compared. Twenty-five selected VASARI features and five other MRI features, including infratentorial involvement, coexistence of both infratentorial and supratentorial involvement, enhancement pattern, notch sign, and butterfly sign were evaluated in preoperative brain MRIs of 31 patients (Table 1). Non-enhancing tumors (nCET) are a rare finding in CNS lymphomas [20, 21]. Peritumoral edema and nCET can sometimes be radiologically indistinguishable. In evaluating F6, F13, and F22 features associated with nCET from the VASARI set, as well as in distinguishing edema, FLAIR and T2 images were used in addition to C + T1 images. Signal changes limited to the white matter, with preservation of cortical and deep gray matter structures, a concentric increase in T2 signal around the enhancing tumor, higher T2 signal, and limited or absent mass effect were considered indicators of peritumoral edema. In contrast, FLAIR signal increases involving deep gray matter structures and cortex, a more infiltrative pattern, an eccentric increase in T2 signal around the enhancing tumor, lower T2 values, and the presence of mass effect were evaluated as nCET [22]. The enhancement pattern was evaluated in four categories as stated in the study of Senocak et al. as follows: homogenous nodular, perivenular, patchy and ring-like [3]. The notch sign, which was defined as an abnormally deep depression at the tumor margin in contrast-enhanced MRI in PCNSL, was evaluated in both PCNSL and SCNSL groups (Fig. 2) [23]. Another MRI feature evaluated was the butterfly pattern. Although the butterfly pattern was first described for glioblastomas affecting both hemispheres by crossing the corpus callosum, it has been reported that this pattern is also seen in CNS lymphomas [8, 24].

**Table 1:** Summary of VASARI and other MRI features

Number	Name	Options
F1	Tumor location	Frontal; Temporal; Insular; Parietal; Occipital; Brainstem, Cerebellum, Basal ganglia, Thalamus, Corpus callosum
F2	Side of tumor epicenter	Right; Center/Bilateral; Left
F3	Eloquent Brain	None; Speech motor; Speech receptive, Motor, Vison
F4	Enhancement quality	None; Mild; Marked
F5	Proportion enhancing	N/A; <5%; 6-~33%; 34 ~ 67%; 68 ~ 95%; >95%
F6	Proportion non-enhancing	N/A; <5%; 6-~33%; 34 ~ 67%; 68 ~ 95%; >95%
F7	Proportion necrosis	N/A; <5%; 6-~33%; 34 ~ 67%; 68 ~ 95%; >95%
F8	Cyst	No; Yes
F9	Multifocal or multicentric	Single; Multifocal; Multicentric; Generalized
F10	T1/FLAIR ratio	Expansive; Mixed; Infiltrative
F11	Thickness of enhancing margin	Thin; Thick; Solid
F12	Definition of enhancing margin	N/A; Well-defined; Poorly-defined
F13	Definition of non-enhancing margin	N/A; Well-defined; Poorly-defined
F14	Proportion of edema	N/A; <5%; 6-~33%; 34 ~ 67%; 68 ~ 95%; >95%
F15	Edema crosses midline	No; Yes
F16	Hemorrhage	No; Yes
F17	Diffusion	No ADC image; Facilitated; Restricted; Mixed
F18	Pial invasion	No; Yes
F19	Ependymal invasion	No; Yes
F20	Cortical involvement	No; Yes
F21	Deep white matter invasion	No; Yes
F22	Non-enhancing tumor crosses midline	N/A; No; Yes
F23	Enhancing tumor crosses midline	No; Yes
F24	Satellites	No; Yes
F25	Calvarial remodeling	No; Yes
O1	Coexistence of both infratentorial and supratentorial involvement	No; Yes
O2	Infratentorial involvement	No; Yes
O3	Enhancement pattern	Homogenous nodular; Perivenular; Patchy; Ring-like
O4	Notch sign	No; Yes
O5	Butterfly Sign	No; Yes

VASARI = Visually Accessible Rembrandt Images, N/A = Not applicable

Image evaluation was performed by two radiologists (Y.K.C, K.A.S, with seven and five years of neuroradiology experience, respectively). The readers were blinded to the clinical information and type of lymphoma. The findings of each reader were recorded to assess inter-reader agreement. Afterwards, all images were evaluated under the supervision of a senior neuroradiologist with 26 years of experience and consensus was reached.

### Statistical analysis

The data were analyzed using the IBM SPSS Statistics Standard Concurrent User V 26 (IBM Corp., Armonk, New York, USA) statistical package program. Normality of the data for numerical variables was evaluated using the Shapiro-Wilk normality test. Homogenity of variances was evaluated using the Levene test. Independent samples t-test was used for two-group comparisons of numerical variables in the case of normal distribution of data, and Mann-Whitney U test was used in the absence of normal distribution. Relationships between categorical variables were analyzed using Fisher’s Exact Test and Chi-square test. Since the data on interrater agreement were categorical, they were tested using the Kappa statistic. A p-value < 0.05 was considered statistically significant. Values close to 1 indicate high interrater agreement for that particular feature, whereas values close to 0 signify that interrater agreement is due to chance (0.01–0.20 slight agreement; 0.21–0.40 fair agreement; 0.41–0.60 moderate agreement; 0.61–0.80 substantial agreement; 0.81-1.00 almost perfect agreement).

## Results

### Demographic data and subtypes of lymphoma of the patients

Among the 31 patients, 9 (29.03%) were male, and 22 (70.97%) were female. The mean age of the patients was 56.9 (range: 20–75 ± 13.33). Table 2 presents all demographic data.

**Table 2:** Demographic data of the patients

	Study groups		Test Statistics	
	PCNSL *n =* 19	SCNSL *n =* 12	Test value	*p*
**Sex**, *n* (%)				
Male	5 (26.3)	4 (33.3)	0.176 ^†^	0.675
Female	14 (73.7)	8 (66.7)		
**Age**, (*year*)				
$\:\stackrel{-}{x}$±ss	57.05 ± 12.22	56.67 ± 14.09	0.077 ^‡^	0.939
*M* (*min*-*max*)	58 (21–75)	61.5 (20–71)		
**Lymphoma type**, *n* (%)				
Diffuse large B-cell lymphoma (DLBCL)	19 (100.0)	11 (91.7)	1.636 ^†^	0.201
T-cell lymphoma	0 (0)	1 (8.3)		

^†^: Chi-square test (χ²), ‡: Independent samples t-test (t), Summary statistics for numerical data are given as mean ± standard deviation and median (minimum-maximum), for categorical data, the values are presented as count (percentage)

### VASARI feature set and other MRI features

**Fig. 3:**
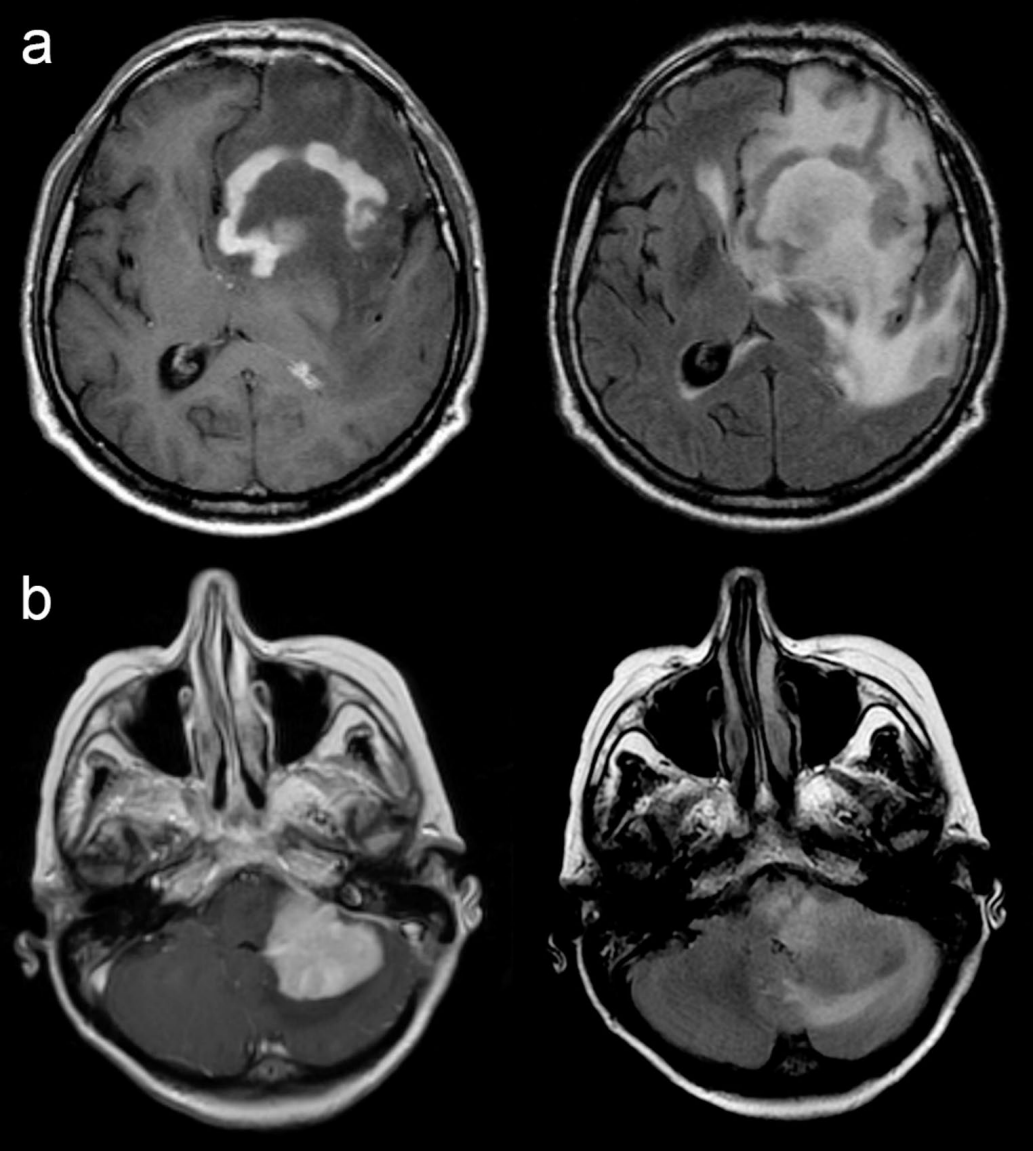
The difference in proportion of edema is depicted in the C + T1W and FLAIR images for a case of PCNSL in the upper row (**a**) and a case of SCNSL in the lower row (**b**). It is noteworthy that there is extensive edema in the PCNSL case, whereas the edema volume is comparatively less in the SCNSL case

No statistically significant difference was observed between the two groups in terms of most VASARI features, except for the tumor localization and proportion of edema. The location of the tumor showed statistically different distribution in PCNSL and SCNSL groups (*p* = 0.036). The most common localization was frontal lobe in the PCNSL, as opposed to corpus callosum and cerebellum in the SCNSL group. In none of the cases, the tumor epicenter was located in the insula, brainstem or thalamus. Proportion of edema was greater in PCNSL group than in SCNSL group (*p* = 0.049) (Fig. 3).

While the presence of infratentorial involvement was observed in three (15.8%) patients in the PCNSL group, it was present in seven (58.3%) patients in the SCNSL group among other MRI features. Although the number of patients with infratentorial involvement was small, there was a statistically significant difference between the groups (*p* = 0.014).

Notch sign was detected in 14 (73.7%) patients in the PCNSL group and in four (33.3%) patients in the SCNSL group; the difference was statistically significant (*p* = 0.027). The statistical comparison of VASARI and other MRI features in differentiating PCNSL from SCNSL are shown in Tables 3 and 4.

**Table 3:** Comparison of VASARI features according to study group

	Study groups (Consensus)				Test Statistics ^†^	
	PCNSL		SCNSL		Test value	p
	*n =* 19		*n =* 12			
**F1 Tumor location**, *n* (%)					**11.139**	**0.036**
Frontal	9	47.4	2	16.7		
Temporal	0	0	2	16.7		
Parietal	3	15.8	0	0		
Occipital	2	10.5	0	0		
Cerebellum	1	5.3	3	25		
Basal ganglia	3	15.8	2	16.7		
Corpus callosum	1	5.3	3	25		
**F2 Side of tumor epicenter**, *n* (%)						
Right	5	26.3	3	25	0.195	> 0.999
Central/bilateral	11	57.9	7	58.3		
Left	3	15.8	2	16.7		
**F3 Elaquent brain**, *n* (%)						
No	14	73.7	7	58.3	0.793	0.447
Yes	5	26.3	5	41.7		
**F3-1 Elaquent zone involvement**, *n* (%)*						
Speech motor	2	10.5	5	41.7	4.286	0.167
Speech receptive	1	5.3	1	8.3	-	-
Motor	1	5.3	0	0	1.111	> 0.999
Visual	3	15.8	1	8.3	1.667	0.524
**F4 Enhancement**, *n* (%)					-	-
Marked	19	100	12	100		
**F5 Proportion enhancing**, *n* (%)						
%6–33	15	78.9	6	50	5.163	0.065
%34–67	4	21.1	3	25		
%68–95	0	0	3	25		
**F6 Proportion non-enhancing**, *n* (%)						
N/A	16	84.2	11	91.7	1.331	0.761
<%5	2	10.5	0	0		
%6–33	1	5.3	1	8.3		
**F7 Proportion necrosis**, *n* (%)						
N/A	10	52.6	7	58.3	1.054	0.607
<%5	6	31.6	2	16.7		
%6–33	3	15.8	3	25		
**F8 Cyst**, *n* (%)					-	-
No	19	100	12	100		
**F9 Multifocality**, *n* (%)						
Single	6	31.6	5	41.7	4.358	0.238
Multifocal	5	26.3	0	0		
Multicentric	7	36.8	5	41.7		
Generalized	1	5.3	2	16.7		
**F10 T1/FLAIR ratio**, *n* (%)						
Expansive	3	15.8	2	16.7	1.314	0.705
Mixed	15	78.9	8	66.7		
Infiltrative	1	5.3	2	16.7		
**F11 Thickness of enhancing margin**, *n* (%)						
Thin (< 3 mm)	0	0	1	8.3	2.339	0.311
Thick ( > = 3 mm)	4	21.1	1	8.3		
Solid	15	78.9	10	83.3		
**F12 Definition of enhancing margin**, *n* (%)					-	-
Well-defined	19	100	12	100		
**F13 Definition of non-enhancing margin**, *n* (%)						
N/A	15	78.9	9	75	0.526	> 0.999
Well-defined	1	5.3	1	8.3		
Poorly-defined	3	15.8	2	16.7		
**F14 Proportion of edema**, *n* (%)					**6.425**	**0.049**
<%5	0	0	1	8.3		
%6–33	1	5.3	4	33.3		
%34–67	5	26.3	3	25		
%68–95	13	68.4	4	33.3		
**F15 Edema crosses midline**, *n* (%)						
No	5	26.3	5	41.7	0.793	0.447
Yes	14	73.7	7	58.3		
**F16 Hemorrhage**, *n* (%)						
No	15	78.9	6	50	2.82	0.093
Yes	4	21.1	6	50		
**F17 Diffusion**, *n* (%)						
No ADC image	5	26.3	5	41.7	2.219	0.42
Restricted	8	42.1	2	16.7		
Mixed	6	31.6	5	41.7		
**F18 Pial invasion**, *n* (%)						
No	9	47.4	5	41.7	0.097	> 0.999
Yes	10	52.6	7	58.3		
**F19 Ependymal invasion**, *n* (%)						
No	8	42.1	3	25	0.94	0.452
Yes	11	57.9	9	75		
**F20 Cortical involvement**, *n* (%)						
No	4	21.1	3	25	0.066	> 0.999
Yes	15	78.9	9	75		
**F21 Deep white matter invasion**, *n* (%)						
No	4	21.1	4	33.3	0.579	0.676
Yes	15	78.9	8	66.7		
**F22 Non-enhancing tumor crosses midline**, *n* (%)						
N/A	16	84.2	11	91.7	0.715	> 0.999
No	2	10.5	1	8.3		
Yes	1	5.3	0	0		
**F23 Enhancing tumor crosses midline**, *n* (%)						
No	11	57.9	10	83.3	2.178	0.24
Yes	8	42.1	2	16.7		
**F24 Satellites**, *n* (%)						
No	12	63.2	8	66.7	0.04	> 0.999
Yes	7	36.8	4	33.3		
**F25 Calvarial remodeling**					-	-
No	19	100	12	100		

^†^: The Chi-square test, Summary statistics presented as *Count (Percentage)*. Sections highlighted in bold are statistically significant (*p* < 0.05)

**Table 4:** Comparison of other radiological features according to study groups

	Study groups (Consensus)				Test Statistics ^†^	
	PCNSL *n =* 19		SCNSL *n =* 12		Test value	*p*
**Coexistence of both infratentorial and supratentorial involvement**, *n* (%)						
No	16	84.2	9	75.0	0.400	0.527
Yes	3	15.8	3	25.0		
**Infratentorial involvement**, *n* (%)						
No	16	84.2	5	41.7	**6.092**	**0.014**
Yes	3	15.8	7	58.3		
**Enhancement pattern**, *n* (%)						
Homogenous nodular	18	58.1	11	64.7	0.115	0.735
Perivenular	4	19.4	2	11.8	0.091	0.763
Patchy	6	12.9	2	11.8	0.854	0.355
Ring-like	3	9.7	2	11.8	0.004	0.948
**Notch sign**, *n* (%)						
No	5	26.3	8	66.7	**4.918**	**0.027**
Yes	14	73.7	4	33.3		
**Butterfly sign**, *n* (%)						
No	15	78.9	10	83.3	0.091	0.763
Yes	4	21.1	2	16.7		

^†^: The Chi-square test, Summary statistics presented as *Count (Percentage)*. Sections highlighted in bold are statistically significant (*p* < 0.05)

### Interrater agreement

The inter-reader agreement for VASARI features ranged from moderate to almost perfect, while for other MRI features, it ranged from fair to almost perfect. The highest inter-reader agreement for VASARI features was observed in multifocality, whereas the lowest agreement was noted in pial invasion. Regarding other MRI features, the highest agreement was achieved in butterfly sign. The inter-reader agreement for all of the features are presented in Table 5.

**Table 5:** Interobserver agreement for VASARI and other MRI features

	Kappa (κ)
F1 Tumor location	0.881
F2 Side of tumor epicenter	0.945
F3 Elaquent brain	0.64
*F3-1 Speech motor*	*0.631*
*F3-2 Speech receptive*	*0.465*
*F3-3 Motor*	*0.999*
*F3-4 Visual*	*0.999*
F4 Enhancement ^ϕ^	*-*
F5 Proportion enhancing	0.727
F6 Proportion non-enhancing	0.582
F7 Proportion necrosis	0.685
F8 Cyst ^ϕ^	-
F9 Multifocality	0.954
F10 T1/FLAIR ratio	0.512
F11 Thickness of enhancing margin	0.649
F12 Definition of enhancing margin ^ϕ^	-
F13 Definition of non-enhancing margin	0.68
F14 Proportion of edema	0.522
F15 Edema crosses midline	0.852
F16 Hemorrhage	0.723
F17 Diffusion	0.807
F18 Pial invasion	0.47
F19 Ependymal invasion	0.933
F20 Cortical involvement	0.903
F21 Deep white matter invasion	0.757
F22 Non-enhancing tumor crosses midline	0.679
F23 Enhancing tumor crosses midline	0.859
F24 Satellites	0.931
F25 Calvarial remodeling ^ϕ^	-
O1 Coexistence of both infratentorial and supratentorial involvement	0.903
O2 Infratentorial involvement	0.784
*O3-1 Homogenous nodular*	*0.367*
*O3-2 Perivenular*	*0.739*
*O3-3 Patchy*	*0.385*
*O3-4 Ring-like*	*0.762*
O4 Notch sign	0.741
O5 Butterfly sign	0.999

^ϕ^: Since both readers made identical decisions, inter-reader agreement was not assessed

## Discussion

In our study we found that MRI findings of parenchymal involvement of primary and secondary CNS lymphomas were quite similar. However, tumor localization and proportion of edema in the VASARI feature set and infratentorial involvement and notch sign in other MRI features were significantly different between PCNSL and SCNSL.

We observed a predilection of PCNSL to predominantly involve the supratentorial compartment, especially frontal lobe (47.7%). Other studies reported frontal lob location in 20-49% of PCNSL patients, consistent with our results [7, 25]. Infratentorial involvement was present in 58.3% of patients with SCNSL and 15.8% of patients with PCNSL in our cohort. Previous studies have reported infratentorial involvement in SCNSL ranging from 16.7 to 42.9%, while in PCNSL, it has been reported between 34.6% and 50% [3–5, 26]. Compared to previous studies, infratentorial involvement of SCNSL was more common in our series. Among the SCNSL cases involving the infratentorial compartment, four had isolated infratentorial involvement, while three cases showed concomitant supratentorial involvement. Although infratentorial involvement is a more commonly expected finding in SCNSL, it can also be observed, though less frequently, in PCNSL.

To the best of our knowledge, the presence of the notch sign has not been reported previously in the differentiation of PCNSL from SCNSL. In our series, 73.7% of cases with PCNSL demonstrated the notch sign, whereas only one-third of cases with SCNSL showed this finding. The notch sign was defined as an abnormal deep indentation at the tumor margin on contrast-enhanced MR images of PCNSL patients [23]. In the study of Mansour et al., it was stated that the PCNSL occurs subcortically, respecting the adjacent cortex and its morphology, resulting in a crescent appearance [27]. We believe that the notch sign and crescent appearance represent the same radiological manifestations of equivalent morphological alterations. However, there are no studies examining either the notch sign or the crescent appearance in the SCNSL. While the notch sign serves as a valuable aid in differentiating between PCNSL and SCNSL, it is important to bear in mind that it may also be present in cases of SCNSL. We also observed that in cases with notch sign, this feature was exclusively caused by a tumor located in the supratentorial compartment. In our series, infratentorial involvement of SCNSL was more frequent than that of PCNSL. The cerebellum, which occupies a large portion of the infratentorial compartment, has a higher neuronal density and a more compact anatomical structure compared to the cerebrum [28, 29]. Since lymphoma is known as a plastic tumor, it can be hypothesized that the lower frequency of notch sign in SCNSLs is related to the compact structure of the cerebellum. However, this hypothesis needs to be investigated in larger case series.

Scoring of VASARI features which estimate proportion requires assesment of the entire MRI study. It is assumed that the entire signal abnormality consists of four different components: enhancing tumor, non-enhancing tumor, necrosis, and edema. We found that proportion of vasogenic edema was significantly higher in the PCNSL group. This finding, which seems to be helpful in differentiating PCNSL from SCNSL, has not been previously described. Compared to glioblastoma, PCNSL showed a higher degree of blood-brain barrier disruption within the tumor mass resulting in increased vascular permeability. Neoplastic lymphoid cells can easily expand the vascular wall by penetrating through the rich reticulin fiber network [30]. This blood-brain barrier disruption in the tumor mass is seen as increased vascular permeability and could correspond to vasogenic edema on MRI. In our study, the relatively higher occurence of multifocality/multicentricity and greater degree of perivenular contrast enhancement observed in cases of PCNSL compared to SCNSL may offer an explanation for the higher proportional prevalence of edema in this particular group. However, it is important to note that the higher peritumoral edema observed in the PCNSL group may not be generalizable due to the limited sample size and the history of prior steroid treatment in patients with SCNSL, where the shortest interval between imaging and steroid treatment was approximately three weeks in our cohort. These factors necessitate caution in interpreting the peritumoral edema difference and their applicability to broader patient populations. There is a also a need for dynamic contrast-enhanced MR studies evaluating vascular permability parameters such as *K*^trans^, K_ep_, and V_e_ to elucidate why the proportion of edema is higher in patients with PCNSL compared to SCNSL.

**Fig. 4:**
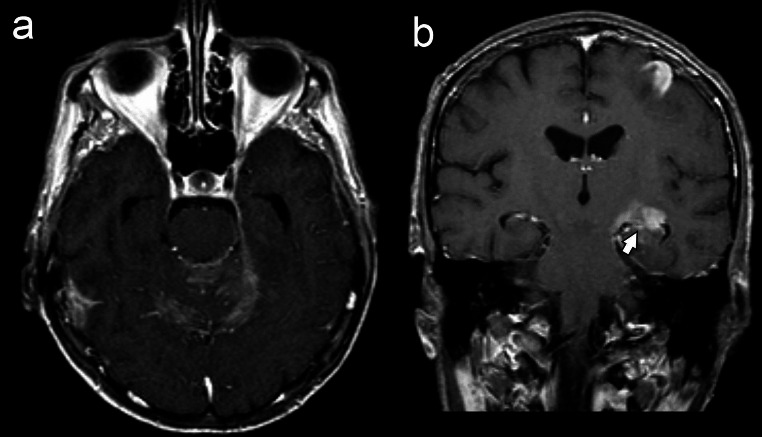
An axial (**a**) C + T1W image of a 61-year-old male patient with SCNSL shows cerebellar leptomeningeal enhancement. The coronal C + T1W image (**b**) reveals a parenchymal lesion in the left frontal and temporal lobes, along with ependymal enhancement in the ipsilateral temporal horn (arrow)

Hill et al. reported parenchymal involvement in only one third of cases with SCNSL in their review. However, in most of the articles reviewed in this article, the diagnosis was made by cerebrospinal fluid examination or CT findings or clinical data that were not radiologically correlated [31–34]. Parenchymal involvement may not have been detected in these studies since MRI evaluation was not performed. In contrary to these studies, parenchymal lesions were observed in all SCNSL cases in our series. 7/12 SCNSL cases had pial involvement and 9/12 had ependymal involvement. (Fig. 4). Similar to our study, Malikova et al. reported analyzing the MRI appearances of 21 patients with SCNSL, parenchymal involvement was reported in all but one patient [5]. Mass-forming parenchymal brain involvement has been reported as an uncommon form of invasion into the CNS for systemic lymphomas [35]. However, we found no significant difference in the type of parenchymal involvement (mass-forming versus pial/ependymal) between PCNSL and SCNSL.

In our study, almost perfect inter-reader agreement was observed in 9 out of the 25 VASARI features. Substantial agreement was achieved in 8 features, while moderate agreement was observed in 4 features. In the remaining four VASARI features, both readers made identical decisions. Both readers assessed contrast enhancement as marked and well-defined, while neither cyst nor calvarial remodelling was detected in any case. In a study evaluating the inter-reader agreement of VASARI features in pediatric brain tumors, acceptable agreement was found in 7 out of the first 25 features. Features with substantial agreement and almost perfect agreement were considered acceptable [36]. In a recent study by Setyawan et al.‘s, which utilized VASARI features for the prediction of grade and molecular parameters of gliomas, the inter-reader agreement was found to be between the kappa coefficent of 0.714 and 0.831 [37]. Given the high inter-reader agreement achieved in our study, we can assume that VASARI feature guide as well as other specific features can be used in the assessment of CNS lymphomas.

There are several limitations of the present study. The primary limitation of our study is the small sample size, which limits the broader relevance of our findings. Larger studies investigating the morphological MRI features of CNS lymphomas are needed to further validate our results. The variability in MRI protocols was due to the wide temporal scope encompassed by the retrospective study design. Therefore, diffusion-weighted images and susceptibility-weighted images were not obtained in some of the cases. Another limitation of the study is the visual assessment of the VASARI feature set rather than quantitative measurements. Additionally, the absence of immunocompromised cases in the evaluated PCNSL group hinders the generalizability of our results.

## Conclusion

Contrast-enhanced MRI plays a crucial role in the diagnosis of CNS lymphoma. Although it can be challenging to distinguish between PCNSL and SCNSL based on imaging findings, certain radiological features may contribute to this differentiation. A greater degree of proportional edema, the presence of the notch sign, and frontal lobe location are indicative of PCNSL, whereas infratentorial involvement supports a diagnosis of SCNSL.
